# A 0.8-cm clear cell neuroendocrine tumor G1 of the gallbladder with lymph node metastasis: a case report

**DOI:** 10.1186/s12957-018-1454-y

**Published:** 2018-07-23

**Authors:** Yuki Hirose, Jun Sakata, Kazuhiko Endo, Masato Takahashi, Reijiro Saito, Hiroshi Imano, Tomoki Kido, Kei Yoshino, Toshiki Sasaki, Toshifumi Wakai

**Affiliations:** 1 Division of Digestive and General Surgery, Niigata University Graduate School of Medical and Dental Sciences, Niigata, 951-8510 Japan; 2 Department of Digestive Surgery, Akita Kousei Medical Center, Akita, Japan; 3 Department of Diagnostic Pathology, Akita Kousei Medical Center, Akita, Japan

**Keywords:** Neuroendocrine tumor, Lymph node metastasis, Gallbladder, Surgical resection

## Abstract

**Background:**

Neuroendocrine tumors (NETs) of the gallbladder are rare and generally considered low-grade malignancies. We herein describe a case of a patient with a 0.8-cm clear cell NET G1 of the gallbladder with nodal involvement.

**Case presentation:**

A 65-year-old man with no medical history indicative of von Hippel-Lindau (VHL) disease underwent laparoscopic cholecystectomy for cholecystolithiasis. There was a 0.8-cm tumor in the neck of the gallbladder. Histologic examination revealed nests or trabecular growth of clear cells with small round-to-oval nuclei. Immunohistochemically, tumor cells showed positivity for chromogranin A and synaptophysin; Ki-67 index was < 1.0%. Based on the World Health Organization 2010 classification, we made a diagnosis of clear cell variant of NET G1 without VHL disease. The tumor invaded the muscular layer and had no extension to the perimuscular connective tissue but had metastasized to a cystic duct node. A radical second resection with regional lymphadenectomy of the gallbladder was performed, and there was no metastasis on histology. After the definitive surgery, he was followed up for 10 months without adjuvant therapy and is alive and well with no evidence of recurrence.

**Conclusions:**

Our experience suggests that, even when smaller than 1 cm, NET G1 of the gallbladder can metastasize. When NET G1 is incidentally identified in the gallbladder of a surgical specimen, detailed pathologic examination of the cystic duct node, when found, should be performed to guide whether a radical second resection with regional lymphadenectomy is appropriate.

## Background

Neuroendocrine tumor (NET) is traditionally an umbrella term that encompasses a broad family of neoplasms originating from neural and endocrine structures located all over the body, especially in the gastrointestinal tract and lung [1, 2]. Primary NETs of the gallbladder are rare and constitute < 1% of all NETs [1, 3]. Clear cell NET of the gallbladder is even rarer; it has been described in association with von Hippel-Lindau (VHL) disease [4], which is a rare autosomal hereditary disease and generates tumors of the pancreas, kidneys, central nervous system, adrenal glands, and reproductive organs, but rarely of the biliary system [5].

The World Health Organization (WHO) 2010 classification divides these tumors into NET G1, G2, and G3 (or neuroendocrine carcinoma: NEC), based on the histologic evaluation of the proliferation rate and mitotic index of NET cells [6]. This classification enables prognostically relevant patient stratification, and NET G1 is thought to be a relatively “benign” category. Also, the risk of malignant behavior of NETs is widely accepted to increase in a size-dependent fashion [6–8]. Here, we describe a case of a patient who had a tiny clear cell NET G1 of the gallbladder with nodal involvement.

## Case presentation

**Fig. 1:**
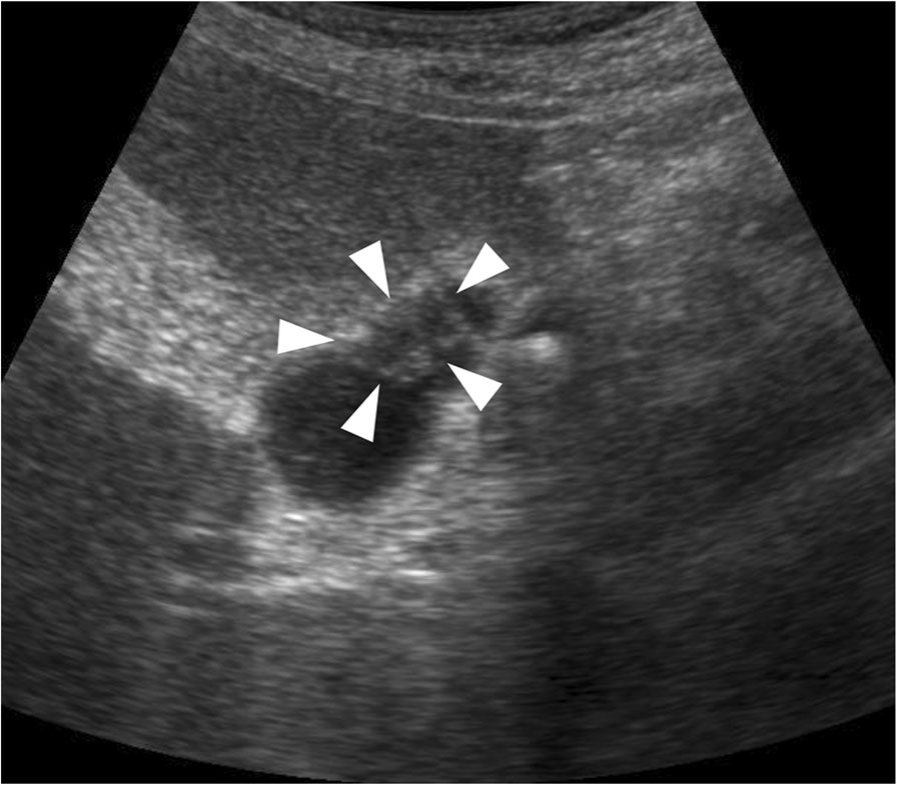
Ultrasonography image shows a 0.8-cm low echoic nodule (arrowheads) in the neck of the gallbladder, which was preoperatively suspected to be a Rokitansky-Aschoff sinuses

A 64-year-old man presented to our hospital with epigastric pain. He exhibited no hormone-related symptoms, such as flushing, diarrhea, stomach aches, or hypoglycemia, and had no past or family history of VHL disease. On admission, the abdomen was nontender and laboratory data showed mild elevation of liver enzymes (AST, 474 IU/L; ALT, 231 IU/L) and the white blood cell count (11 × 10^3^/μL). Abdominal ultrasonography showed the gallbladder and common bile duct stones and a low echoic nodule in the neck of the gallbladder preoperatively suspected as Rokitansky-Aschoff sinuses (Fig. 1). Contrast-enhanced abdominal computed tomography (CT) also revealed gallbladder and common bile duct stones but did not reveal a mass in the neck of the gallbladder. The patient underwent endoscopic sphincterotomy, and common bile duct stones were extracted successfully.

**Fig. 2:**
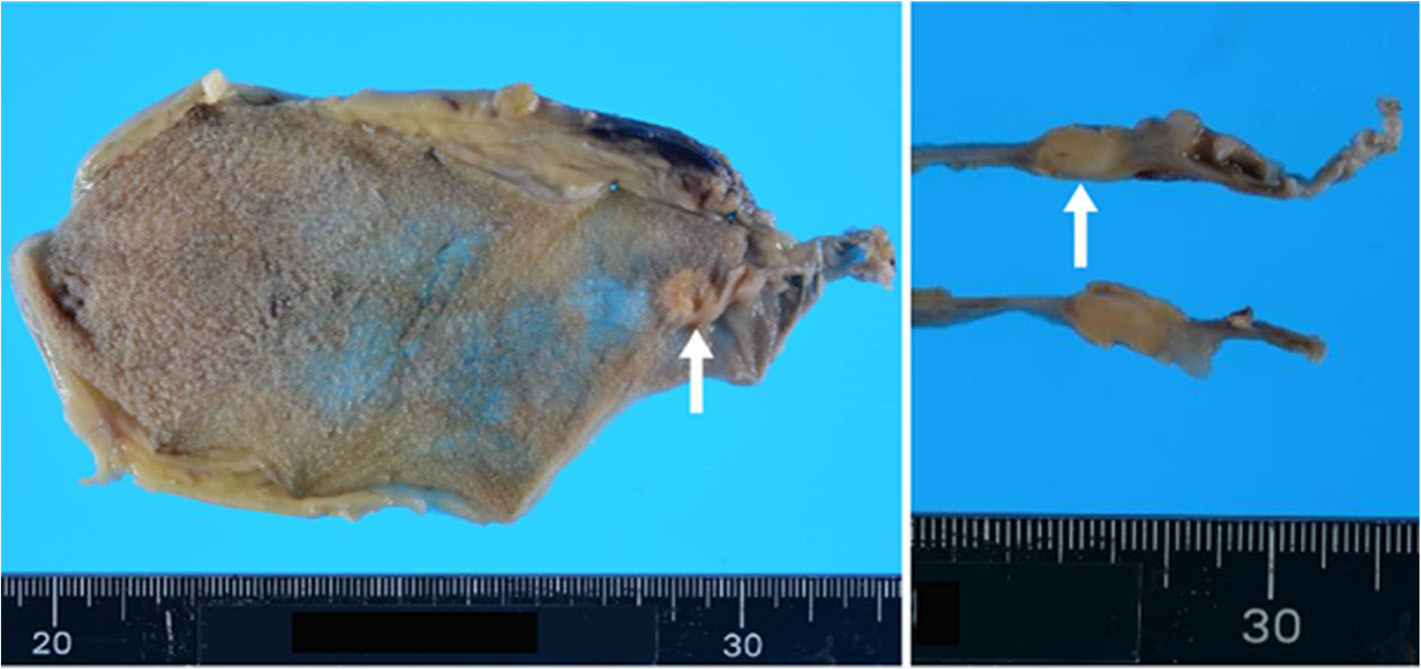
Surgically resected specimen: a yellowish submucosal tumor (0.8 × 0.8 cm) located in the neck of the gallbladder

**Fig. 3:**
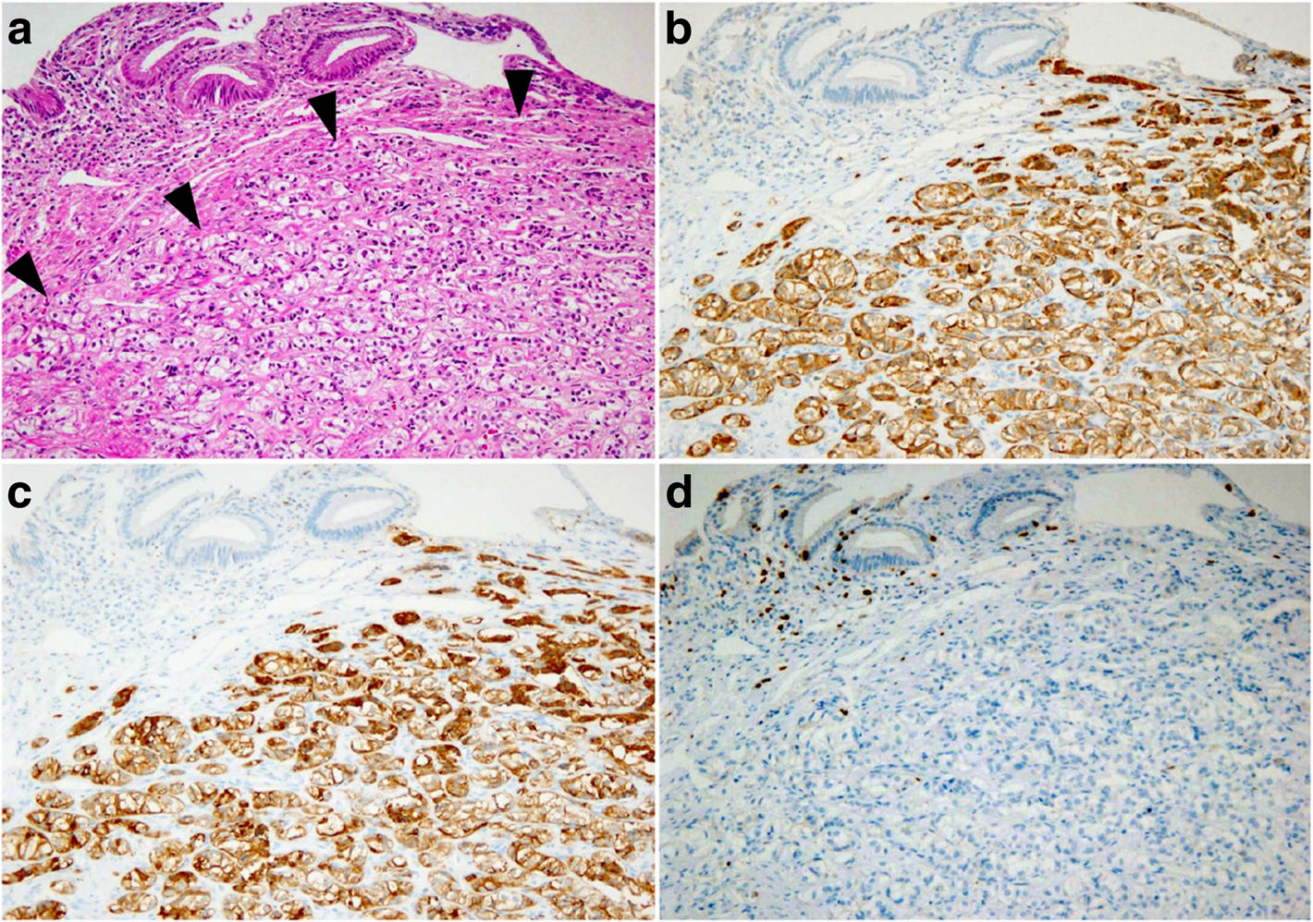
**a** Hematoxylin and eosin staining. Tumor cells (arrowheads) show nests or trabecular growth, have rich clear cytoplasm and small round-to-oval nuclei with inconspicuous nucleoli, and had no mitosis. **b** Immunohistochemical staining of chromogranin A. Tumor cells show positive expression. **c** Immunohistochemical staining of synaptophysin. Tumor cells show positive expression. **d** Immunohistochemical staining of Ki-67. Tumor cells show Ki-67 index < 1.0%. (**a**–**d**: original magnification × 100)

Almost 1 month after endoscopic treatment, laboratory data were within normal limits. We did not check the levels of tumor marker, urinary 5-hydroxyindoleacetic acid, or plasma serotonin because neither cancer nor NET was suspected at that time. He underwent laparoscopic cholecystectomy. Macroscopically, the specimen contained a yellowish submucosal nodule, located in the neck of the gallbladder, the size of which was 0.8 × 0.8 cm (Fig. 2). Histologic examination revealed nests or trabecular growth of clear cells containing small round-to-oval nuclei with inconspicuous nucleoli (Fig. 3a) and showing no mitosis. The tumor surface was covered by intact epithelium. Immunohistochemically, tumor cells showed the expression of chromogranin A (Fig. 3b) and synaptophysin (Fig. 3c), and a Ki-67 index < 1.0% (Fig. 3d). Pathologic diagnosis of the tumor was NET G1 according to the WHO 2010 classification and a clear cell variant without VHL disease. The tumor invaded the muscular layer and showed no extension to the perimuscular connective tissue, but had metastasized to a cystic duct node.

A radical second resection with regional lymphadenectomy of the gallbladder was performed, and histologically there was no metastasis. An R0 resection was confirmed, and he received no adjuvant therapy. After discharge, he was followed up every 1–3 months on an outpatient basis with physical examination, laboratory tests, and/or contrast-enhanced abdominal CT, which showed no evidence of recurrence. He remains alive and well 10 months after the definitive resection.

## Discussion

Oberndorfer first described NETs and coined the term “carcinoid” (or “Karzinoide”) in 1907 for these “presumptively” benign neoplasms [9]. Primary NETs of the gallbladder were first described by Joel in 1929. The majority of primary NETs occur in the gastrointestinal tract (66%) [10], followed by the bronchopulmonary system (31%), and then less frequently by locations including the pancreas, hepatobiliary system, ovaries, and testes [10]. The gallbladder is a rare site for NETs, accounting for only 0.04–0.5% of all NETs [5, 11].

In gastroenteropancreatic NETs, it is known that the risk of metastasis largely depends on tumor size [12–14]. As for the gallbladder, Albeores-Saavedra et al. [15] reported that carcinoid tumors of the gallbladder measuring less than 0.5 cm had no clinical significance. Porter et al. [16] reported that only 6% of carcinoid tumors of the gallbladder smaller than 1 cm metastasized, whereas 70% of tumors 2 cm or larger were associated with metastasis. Yokoyama et al. [17] reported that two of seven cases (28.6%) of carcinoid tumors less than 1 cm had metastasis at presentation, whereas all five cases measuring 3 cm or more had metastasis. Although the histologic definition of carcinoid tumor differs from that of NETs, these data indicate that the metastatic risk of NETs of the gallbladder is also size-dependent.

The WHO 2010 classification divides NETs into three categories according to mitotic count and Ki67 labeling index: well-differentiated, low-grade (NET G1); well-differentiated, intermediate-grade (NET G2); and poorly differentiated, high grade (NET G3 or NEC). This classification enables prognostically relevant patient stratification, and NET G1 is thought to be a relatively “benign” category. However, in our case, a tiny gallbladder NET G1 was associated with nodal involvement. To the best of our knowledge, this is the first report that describes a case of gallbladder NET G1, smaller than 1 cm, with nodal involvement. Our experience suggests that, even when smaller than 1 cm in diameter, NET G1 of the gallbladder can metastasize.

Preoperative diagnosis of NETs of the gallbladder is rare because, in general, the patients present with non-specific symptoms such as upper abdominal pain, discomfort, jaundice, and weight loss [18]. Previous studies showed that few cases of gallbladder NETs manifested with carcinoid syndrome [16, 19]. Due to the lack of specific symptoms, the majority of NETs of the gallbladder are identified incidentally after cholecystectomy for cholecystolithiasis [19]. When they are identified in the gallbladder upon removal for presumed benign disease, detailed pathologic examination of the cystic duct node, when found, should be performed to guide whether a radical second resection with regional lymphadenectomy is appropriate. In cases with metastasis in cystic duct nodes, like ours, radical second resection with lymph node dissection should be considered as an option for accurate nodal evaluation, based on the reported outcomes of incidental gallbladder cancer [20].

NET cells have eosinophilic to amphophilic cytoplasm. Rare cases, however, have rich clear cytoplasm and are described as clear cell variants. The clear cell NETs of the pancreas have been reported to be a manifestation of VHL disease [21]. Only three cases of clear cell variant of gallbladder NETs have been described in the literature, one associated with VHL disease [4] and the others not associated with it [22, 23]. Our case is the third reported case in the literature that describes a clear cell NET of the gallbladder without VHL disease. It would appear that clear cell NETs of the gallbladder are not closely associated with VHL disease.

## Conclusions

Gallbladder NET G1, even when smaller than 1 cm, can metastasize. When NET G1 is incidentally identified in a gallbladder surgical specimen, detailed pathologic examination of the cystic duct node, when found, should be performed to guide whether a radical second resection with regional lymphadenectomy is appropriate.

## Abbreviations

CT: Computed tomography
NET: Neuroendocrine tumors
VHL: Von Hippel-Lindau
WHO: World Health Organization
